# A Novel Sodium Alginate-Carnauba Wax Film Containing Calcium Ascorbate: Structural Properties and Preservative Effect on Fresh-Cut Apples

**DOI:** 10.3390/molecules28010367

**Published:** 2023-01-02

**Authors:** Ximeng Lin, Hanyu Zhang, Xi Guo, Yimin Qin, Peili Shen, Qiang Peng

**Affiliations:** 1 College of Food Science and Engineering, Northwest A&F University, Xianyang 712100, China; 2 Key Laboratory of Seaweed Fertilizers, Ministry of Agriculture and Rural Affairs, Qingdao Brightmoon Seaweed Group Co., Ltd., Qingdao 266400, China

**Keywords:** composite edible film, characters, preservation

## Abstract

In order to improve the mechanical properties, nutritional value and fresh-keeping ability of conventional sodium alginate edible composite membranes, a new type of edible composite film was prepared by adding water-blocking agent carnauba wax, plasticizer glycerin, antioxidant and nutritional enhancer sodium ascorbate on a basis of traditional sodium alginate composite film. In this study, the physical, mechanical and structural properties of different film components were investigated. The results showed the components did not simply combine, but produced interaction forces which improved the stability and mechanical properties of composite film. When the amount of calcium ascorbate was 0.4%, the water vapor transmittance of the composite film reached a minimum of 0.65 g·mm/(cm^2^·d·kPa), and the tensile strength and elongation at break reached the maximum, which were 398.64 MPa and 17.93%, respectively. Additionally, the sodium alginate-carnauba wax film exhibited better performance on the preservation of fresh-cut apples. Compared with other composite films, the color and hardness of fresh-cut apples coated with this composite film were better maintained, and the losses of titration acid content and soluble solid content were reduced. Moreover, the weight loss rate, increase in polyphenol oxidase activity and total colony count were inhibited. All results determined that the edible film has good application value in the field of fresh-cut fruit preservation, which provides a theoretical basis for further research on edible film.

## 1. Introduction

Food packaging is a necessary part of food production. It can provide food products with protection from physical, chemical, biological and other external damage in the process of transportation, storage and sales, helping maintain the quality of the food products themselves [1]. Nowadays, due to problems of environmental pollution, energy consumption and food safety caused by traditional plastic packaging, it is imperative to find a green, safe and environmentally-friendly food packaging material for replacement [2]. Environmental and renewable edible film has become a new and attractive material in the field of food packaging [3]. Edible film, which is formed by edible natural polymer materials as raw materials through the interaction of different molecules, has a porous network structure and a certain packaging protection function [4]. According to different raw materials, it can be divided into polysaccharides, proteins, lipids and composite edible films [5]. In recent years, edible films have been widely used in the storage and preservation of fresh-cut fruits due to their simple operation, low cost and good preservation effect. Functionally edible films can be achieved by adding different functional ingredients to edible films, such as antibiotics, antioxidants, edible flavors and probiotics. In this way, it can not only extend the shelf life of food, but also improve its nutritional value. Previous research in this field found that single-component edible film usually has certain weaknesses in practical performance [6]. Hence, it is better to use two or more natural macromolecule substances as the film materials, such that each component can both improve its strengths and complement the functions of the other component(s) in order to obtain a better-performing edible film compared with single-component edible film [7]. Therefore, we often choose two or more natural macromolecule substances as the membrane materials, with each component featuring strengths and circumvented weaknesses. The molecular interaction works to complement each component’s functions in order to obtain a composite edible membrane with better performance.

Sodium alginate is a kind of biological polysaccharide extracted from brown algae. It usually has a linear structure containing β-D-mannonic acid (M) and α-1-guluronic acid (G) with 1–4 glycosidic bonds [8]. Because of its good gel properties and film forming capacity, sodium alginate is often used as edible film material. It can form a film on the surface of food which has certain gas selection permeability. In this case, it can prevent food and external gas exchange to avoid oxidation of the food. In addition, it can protect food from external microorganisms which can create an almost-closed independent environment to keep food fresh. However, the film made by sodium alginate alone is moisture-sensitive due to the strong hydrophilic characteristics of sodium alginate itself, and thus needs to be mixed with other materials [6]. Carnauba wax is extracted from the leaves of the carnauba tree. It is a type of hard and brittle wax with light yellow or brown powder and flake shape. It is mainly composed of cinnamic acid diesters and fatty esters with high melting point and low solubility. These characteristics makes carnauba wax more stable and it has excellent water vapor barrier properties, which have been widely recognized. When mixed, carnauba wax and sodium alginate should complement each other and become an edible composite film with better performance [9]. Glycerol, which is the most widely-used plasticizer in edible membranes [10,11,12], is a small molecule compound to reduce the connection and total cohesive force between the polymer groups and the polymer chain molecules by combining with the polymer groups of the film-forming material in the form of non-bonding [13,14], so that the film becomes soft and flexible to avoid membrane cracks, and the outer appearance of the film is more attractive [15,16]. Calcium ascorbate is a safe and effective browning inhibitor and antioxidant for fruits and vegetables, which can maintain the color and natural flavor of fresh-cut products, extend their shelf life, and rarely has negative effects on them [17,18]. Fresh-cut fruits are convenient, fast and nutritionally safe, and are favored by consumers all over the world [19]. However, compared with intact fresh fruits and vegetables, fresh-cut fruits and vegetables are more prone to physiological aging and moisture and nutrient loss, and discoloration of sliced surfaces, soft texture, and serious microbial infestation can greatly shorten the shelf life of these products [20]. Thus, a composite film with good comprehensive performance is required. However, current research has paid greater attention to the application of edible film, while ignoring research on the properties and structure of the films, as well as the properties-effect relationship [21].

In this study, edible films were prepared by single or mixed sodium alginate, carnauba wax, glycerol and calcium ascorbate. The physical and mechanical properties, structure and compatibility of each film were investigated. In addition, the fresh-cut apples were used to evaluate the preservative effect of each film. The results were determined by the physiological and biochemical changes during storage, which can provide some ideas and methods for the deep study of edible film and preservation of fresh-cut fruit.

## 2. Results and Discussion

### 2.1. Performance of Film

Most polysaccharide-based films are hydrophilic materials, thus increasing water resistance of the films has recently attracted scientists [22]. When combined with other additives which can decrease water vapor permeability of the films, the other physical or mechanical properties of the films will be affected [23].

**Figure 1:**
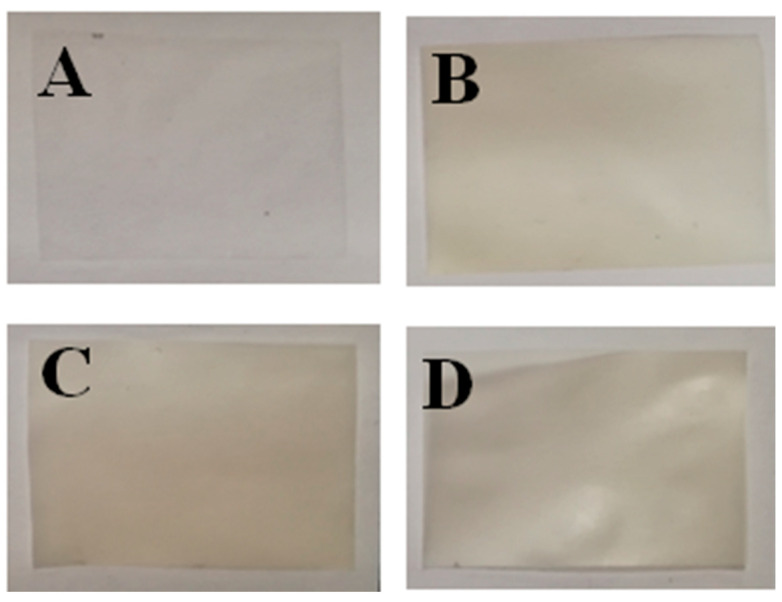
The images of four films (**A**) pure SA membrane; (**B**) SA/CW composite membrane; (**C**) SA/CW/G composite membrane; (**D**) SA/CW/G/Vc-Ca composite membrane).

**Figure 2:**
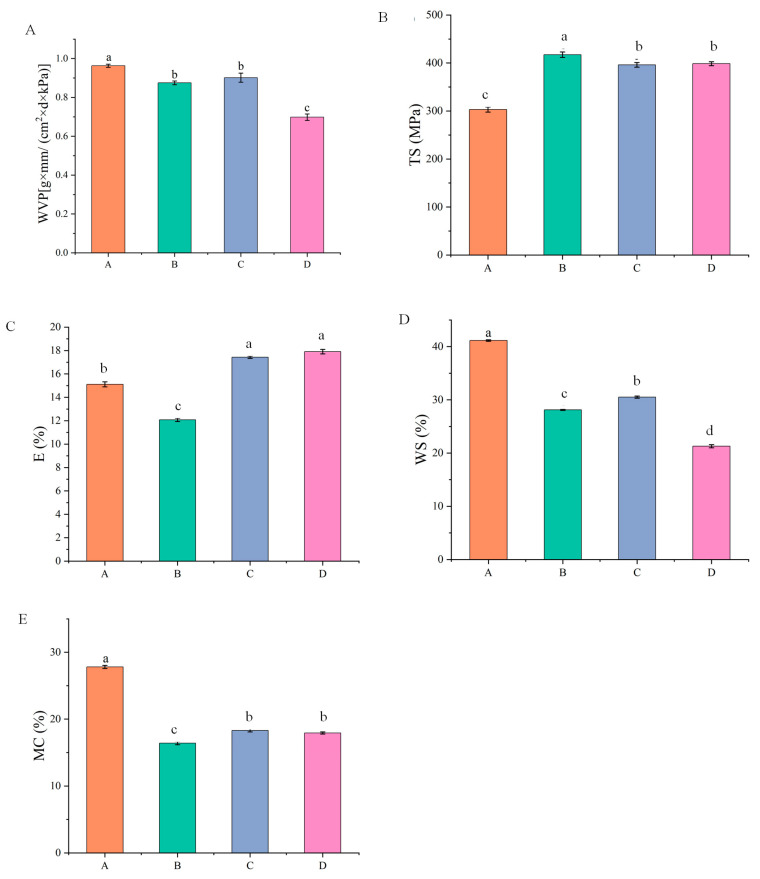
(**A**) Effect of each component on water vapor transmittance of composite film; (**B**) Effect of each component on tensile strength; (**C**) Effect of each component on elongation at break; (**D**) Effect of components on water solubility of composite film; (**E**) Effect of components on water content of composite film (A: pure SA membrane; B: SA/CW composite membrane; C: SA/CW/G composite membrane; D: SA/CW/G/Vc-Ca composite membrane).

The water vapor transmittance of pure SA film, SA-CW composite film, SA-CW-G composite film and SA-CW-G-Vc-Ca composite film was shown in Figure 1 and Figure 2 (Original data shown in Table S1). After adding carnauba wax, the water vapor transmittance decreases, because of the waxy nature of carnauba wax. This result was coincided with that of Galus et al. [24]. However, addition of plasticizer glycerol will make the hydrophilic group of the composite membrane easier to bind with it. Due to the high degree of plasticization, more water molecules passed through the pores of the composite membrane, which resulted in greater water vapor transmittance [25]. When adding calcium ascorbic acid, the Ca^2+^ promotes the cross-linking of the composite membrane, making the structure of the composite membrane more compact [26]. In this case, it is difficult for the water molecules to penetrate, thus reducing the water vapor transmittance which can improve the preservative effect of composite film.

In Figure 2B,C, with the addition of carnauba wax, the tensile strength of the composite film increases, while the elongation at break decreases slightly. The results of film formation experiments showed there will be more cracks after drying the sodium alginate-carnauba wax composite film, caused by the brittleness and hardness of the carnauba wax itself. The negative effect of lipids on the mechanical properties of composite membranes may be attributed to partial replacement of polymers by lipids in the membrane matrix. Spotti [27] observed that with the addition of beeswax to the whey protein concentrate composite membrane, the wax destroys the continuous matrix of biopolymers and forms a non-uniform membrane structure, resulting in a significant decrease in TS and E values. These results were similar to the study conducted by Cecchini [28]. Glycerol, the plasticizer used in the experiment, is a type of low polarity molecule. It can improve the elasticity of the composite film, but its tensile strength is slightly insufficient. Using glycerin and carnauba wax together can apparently improve the mechanical properties of the composite film to a certain extent. In the study of Razavi et al. [29], they determined glycerol can improve flexibility of the film and decrease its tensile strength, which coincided with our results.

The water solubility effect of different components on alginate-based edible film was shown in Figure 2D. It was apparent that the water solubility of composite membrane decreased significantly (31%) with the addition of 0.3% carnauba wax. This is because the hydrophobic groups of carnauba wax cannot interact with water molecules. Therefore, carnauba wax addition can improve the hydrophilic ability of sodium alginate membrane. Additionally, after glycerol was added, the water solubility of the composite film increased because the glycerol molecule contained hydrophilic groups. However, due to the dense structure of the SA-CW-G-Vc-Ca composite film, the internal structure has less pores and the water molecules are difficult to enter, contributing to the water solubility decrease [30]. These results matched with the results of water vapor transmittance of films.

As shown in Figure 2E, the water content of the membrane decreased after adding carnauba wax. This is because the molecules of carnauba wax are mainly hydrophobic and its polarity is small, thus the affinity to water is small, resulting in smaller water content [31]. Glycerol belongs to hydrophilic substance and contains hydroxyl group which can combine water molecules, so that the water content of composite membrane slightly increased after adding it. However, a cross-linking reaction with sodium alginate appeared after the addition of calcium ascorbic acid, leading to an enhanced interaction and the composite membrane structure becoming denser. As a result, the water content of composite membranes significantly decreased compared to pure SA membrane.

### 2.2. Structure of Film

**Figure 3:**
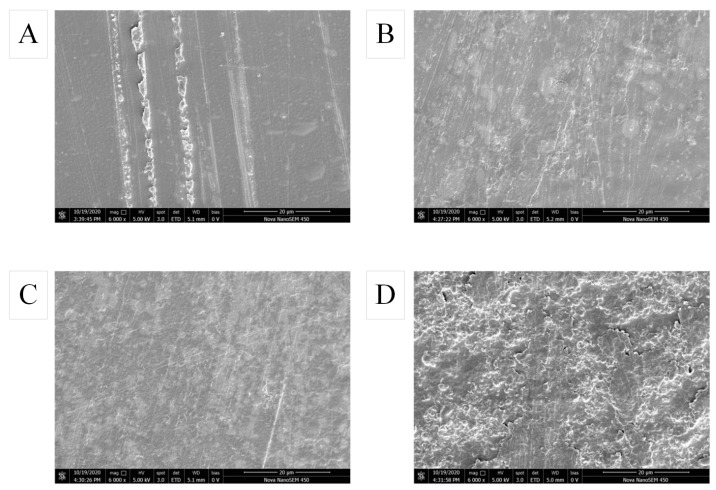
Effect of components on surface morphology of composite film (**A**) pure SA membrane; (**B**) SA/CW composite membrane; (**C**) SA/CW/G composite membrane; (**D**) SA/CW/G/Vc-Ca composite membrane.

SEM is widely used to observe the morphology of packaging surfaces, describe their homogeneity and integrity [32]. The SEM images of pure SA film, SA/CW composite film, SA/CW/G composite film and SA-CW-G-Vc-Ca composite film were shown in Figure 3. In Figure 3A, the surface of the pure sodium alginate film is smooth, and almost no cracks or holes appeared. However, with the addition of carnauba wax, the film’s microstructure significantly changed because of the occurrence of a heterogeneous structure. As shown in Figure 3B, this film had an uneven surface and its surface became rougher. Also apparent was a layered structure formed by wax, resulting in an uneven surface of the film and cracks. This is because the addition of carnauba wax made the structural layer become loose, resulting from the hydrophobicity of carnauba wax (wax drops). Unlike SA-CW composite film, the surface of the film with plasticizer glycerin became smoother because the distribution of carnauba wax is more uniform with fewer wax droplets (Figure 3C). In addition, the cracks were reduced, which also indicates that the carnauba wax was more stable after adding glycerin. The hydrophobic phase has better dispersibility on sodium alginate. As shown in Figure 3D, the surface of the composite film had “protrusions” and the distribution was uniform and dense with the addition of calcium ascorbate, corresponding to the decrease in its water vapor transmission rate. These results were similar to that of Simona et al. [33].

**Figure 4:**
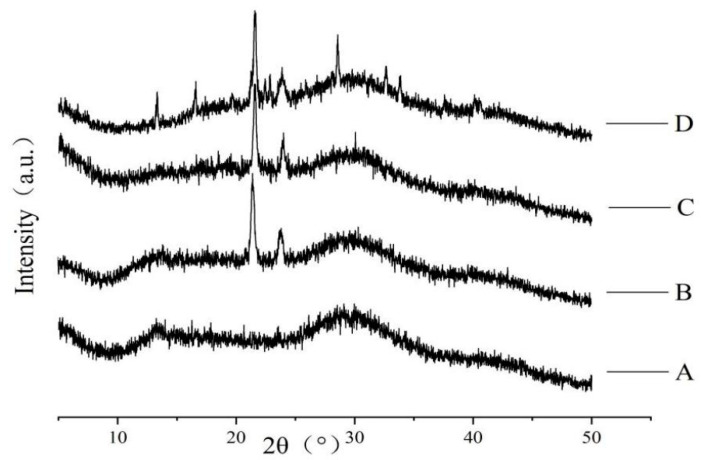
Effect of each component on crystal structure of composite film (A: pure SA membrane; B: SA/CW composite membrane; C: SA/CW/G composite membrane; D: SA/CW/G/Vc-Ca composite membrane).

The influence of each component on the crystal structure of the composite film was obtained by X-ray diffraction analysis. The diffraction spectra of the films is displayed in Figure 4. As the X-ray spectra of pure sodium alginate membrane show, its structure was mainly amorphous and its broad band characteristic peaks at about 2θ = 13° and 2θ = 30°. After adding carnauba wax, the intensity of diffraction peaks decreases at 13° and 30°. This was probably caused by the intermolecular interaction (electrostatic and hydrogen bonding) between the carnauba wax and the alginate, resulting in the dispersion of the carnauba wax molecules into the alginate matrix. At the same time, carnauba wax destroyed the original structure of the sodium alginate matrix. In X-ray diffraction patterns of sodium alginate-carnauba wax composite membrane, two spikes near 2θ = 21° and 2θ = 24° revealed it has crystal structure. With the addition of plasticizer glycerin, a change in peak intensity was observed, but no new peaks appeared. When calcium ascorbic acid was added, the diffraction peaks of the composite film at 2θ = 21° and 2θ = 24° were the smallest, indicating that the intermolecular interaction decreased the crystallinity of the matrix. At the same time, new characteristic peaks appeared at 13°, 16°, 28° and 33°, which was attributed to the cross-linking reaction caused by sodium alginate and Ca^2+^ [34].

**Figure 5:**
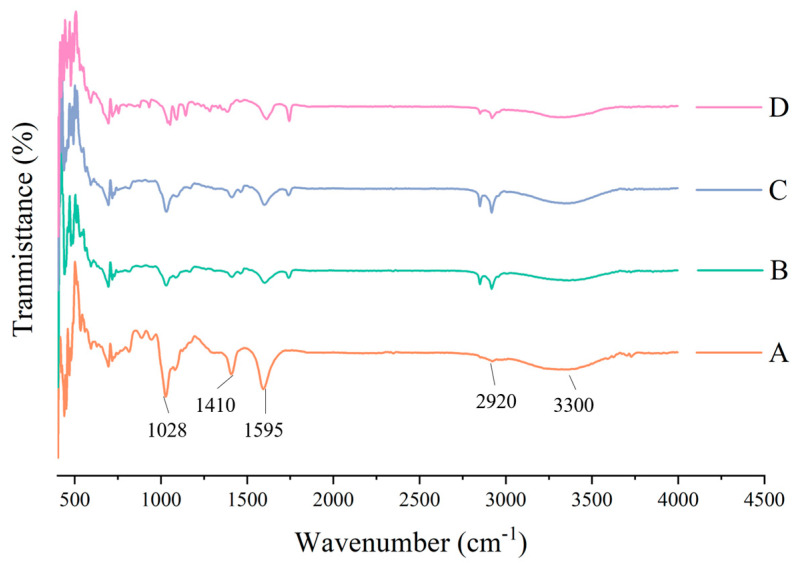
Effect of each component on the chemical bond of composite film (A: pure SA membrane; B: SA/CW composite membrane; C: SA/CW/G composite membrane; D: SA/CW/G/Vc-Ca composite membrane).

The essence of infrared absorption spectrum formation is the rotational-vibrational level transition, and the absorption peak can be determined according to the vibration of the molecule. In Figure 5, in the infrared spectrum of the pure sodium alginate membrane, the peak at 1028 cm^−1^ is the vibration absorption peak of the C-O bond; symmetric and asymmetric expansion vibration of COO^−^ is near 1410 cm^−1^ and 1595 cm^−1^, respectively; the peak at around 2920 cm^−1^ is the telescopic vibration absorption peak of C-H in CH_3_; the spectral band at 3000–3700 cm^−1^ is the telescopic vibration peak generated by the -OH group of sodium alginate, and due to the formation of inter-molecular or inter-molecular hydrogen bonds, the peak shape is wide and blunt. After adding the carnauba wax, at 1724 cm^−1^, a new C=O peak appeared because of the formation of the chemical bonds between carnauba wax and sodium alginate. The CH_2_^−^ absorption peak near 2850 cm^−1^ and the CH_3_ absorption peak strength at around 2920 cm^−1^ increased with the addition of carnauba wax, similar to gelatin films with beeswax and carnauba wax [35]. In addition, the hydroxy band (3000–3700 cm^−1^) is narrower, which showed the hydrophilic reduction of the membrane compared to pure SA films, confirming the results of the WVP. Changes in the wave length and its amplitude band may be associated with the interaction between the sodium alginate and the carnauba wax functional groups [36]. However, absorption strength of 1028 cm^−1^, 1410 cm^−1^, 1595 cm^−1^ and 3300 cm^−1^ peaks became weaker with the addition of carnauba wax. This may be the result of electrostatic and hydrogen bonding between the molecules of the alginate and carnauba wax, which coincides with the results of Gheribi et al. [37]. The spectrum of the composite film with glycerin showed roughly the same main peaks as the sodium alginate-carnauba wax composite film, due to the functional similarity of the low glycerin content. After the addition of calcium ascorbic acid, the COO^−^ absorption peak at 1410 cm^−1^ disappeared and other peaks’ absorption strength weakened, possibly because of a cross-linked reaction of sodium alginate with Ca^2+^. Moreover, the characteristic absorption peaks of pure SA membrane became weaker with the addition of each component, due to the interaction of sodium alginate, carnauba wax, glycerol and calcium ascorbate, resulting in partial compatibility between the components.

### 2.3. Preservative Effect of Edible Composite Film on Fresh-Cut Apples

**Figure 6:**
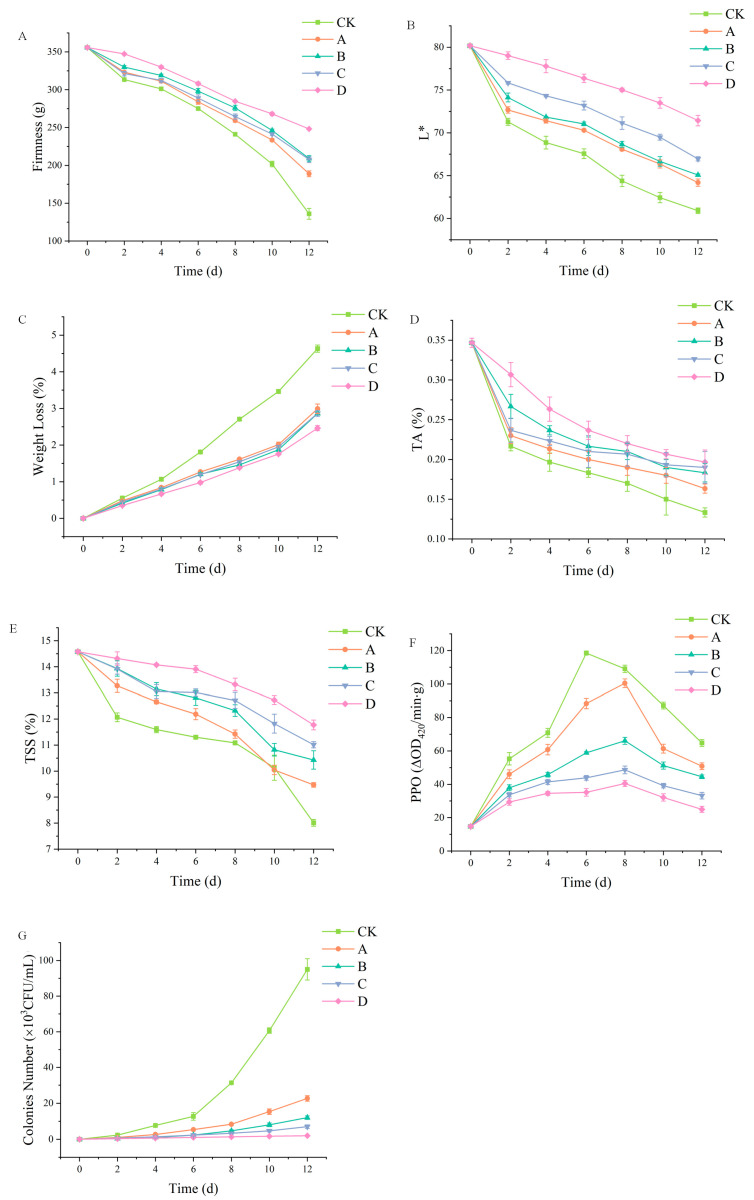
(**A**) Effect of coating treatment on firmness of fresh-cut apple; (**B**) Effect of coating treatment on chroma of fresh-cut apple; (**C**) Effect of coating treatment on weight loss rate of fresh-cut apple; (**D**) Effect of coating treatment on titratable acid of fresh-cut apple; (**E**) Effect of coating treatment on soluble solids of fresh-cut apple; (**F**) Effect of coating treatment on polyphenol oxidase activity of fresh-cut apple; (**G**) Effect of coating treatment on total colonies of fresh-cut apple (A: pure SA membrane; B: SA/CW composite membrane; C: SA/CW/G composite membrane; D: SA/CW/G/Vc-Ca composite membrane). (Original data shown in Table S1).

The application of polysaccharide-based edible films has positive effects on the extension of shelf life of fruits and vegetables and the preservation of their nutritional properties, as well as prevention from microbiology [38,39]. Figure 6A shows the influence of different component membrane materials on the hardness of fresh-cut apples. According to this figure, when the storage time is less than 4 days, the change in the hardness of fresh-cut apples was small. However, as the storage time extended, the hardness of fresh-cut apples gradually decreased. This is because fresh-cut apples’ pectin and cellulose suffered severe water loss at this stage, leading to decreased hardness. The fresh-cut apple hardness drop curves of SA-CW and SA-CW-G composite film solutions were different between pure SA and SA-CW-G-Vc-Ca composite film solutions. On day 12, the hardness of SA-CW-G-Vc-Ca composite membrane liquid film-coated apples was 1.8 times that of the control group. It displayed the least hardness reduction in this experiment, which indicated that composite membrane can effectively alleviate the wilting and wrinkling phenomenon of fresh-cut apples during storage.

Browning is an inevitable phenomenon during the storage of fresh-cut fruit apples. The degree of browning directly affects the appearance quality of fresh-cut fruits and vegetables, and appearance quality is the most direct factor that determines whether consumers purchase the product. Therefore, the color of fresh-cut apple is a more intuitive index to evaluate the fresh-keeping effect. Figure 6B showed L* values of each group decreased gradually with the increase in storage time. However, L* values of the fresh-cut apples treated with pure SA membrane solution, SA-CW membrane solution, SA-CW-G membrane solution and SA-CW-G-Vc-Ca membrane solution were always higher than that of the control group. On day 6, for the control group, the L* value of control group was 67.6, while the L* value of pure SA membrane solution treatment was 70.3 and the L* value of SA-CW-G-Vc-Ca composite membrane solution was 76.4; On day 12, control group fresh-cut apple L* value was 60.9, while the L* value of fresh-cut apple treated with pure SA membrane solution was 64.2% and the L* value of fresh-cut apple treated with SA/CW/G/Vc-Ca composite membrane solution was 71.4%. All these results revealed that the films can effectively inhibit the browning of fresh-cut apples and protect the appearance quality of fresh-cut apple.

As shown in Figure 6C, the weight loss of samples in different treatment groups increased with time. Compared to the control group, each treatment group reduced the weight loss rate of the sample. The quality loss rate of fresh-cut apple in each treatment group was not obvious in the first 4 days and the difference between them was not significant. However, the weight loss rate of fresh-cut apple increased with the increase in storage time. On the 8th day of storage, the weight loss rate of fresh-cut apple in the control group was significantly higher than that in the other treatment groups, at 2.7%. At the same time, the weight loss rates of fresh-cut apple treated with sodium alginate single film, carnauba wax and glycerin were respectively 1.6%, 1.4% and 1.54%, which proved that the addition of carnauba wax can decrease the water vapor transmission of the composite film. Moreover, the weight loss rate of SA/CW/G/Vc-Ca treatment group was only 0.97%, displaying the best density. These results determined that coating treatment could reduce the moisture evaporation of fresh-cut apples, reducing weight loss. In another study, the carboxymethyl cellulose film with propolis extract coating blueberries was observed displaying a similar phenomenon, which demonstrates adding lipids in polysaccharide-based films can improve preservative effect by decreasing its water vapor transmission [40].

Figure 6D showed the titratable acid content of each treatment decreased during the storage period. After 2 days’ storage, the titratable acid content of each treatment group decreased rapidly, probably resulting from the mechanical damage and respiratory consumption of the slices. The coating treatment delayed the decrease in titratable acid content. On the 12th day of storage, the titratable acid content in the SA-CW-G-Vc-Ca composite membrane treatment group was the highest—about 1.3 times that in the control group. The results indicated the coating treatment could effectively maintain the titratable acid content of fresh-cut apple slices.

TSS content, mainly including soluble sugar, acid, cellulose and other components, is the main factor in determining fruit flavor [13]. Effects of different treatment groups on the TSS content of fresh-cut apples were shown in Figure 6E. The content of soluble solids decreased during the whole storage period, and the contents of pure SA membrane, SA-CW membrane, SA-CW-G membrane and SA-CW-G-Vc-Ca membrane were higher than those of the control group. At 0–2 days, the decline was the most obvious, possibly because of some mechanical damage caused by the peeling of apples. As a result, fresh-cut apple tissue cell respiration increased, accelerating the consumption rate of TSS content. On the 12th day of storage, the TSS contents of each treatment group were 8.01%, 9.47%, 10.42%, 11.01%, and 11.8% respectively. Throughout storage, the TSS content of fresh-cut apple treated with SA-CW-G-Vc-Ca composite film solution was always at the highest level of all other groups. In sum, coating treatment can significantly slow down the rate of decrease in the TSS content of fresh-cut apple, and maintain their flavor.

PPO is the most important enzyme that causes browning after the harvesting of fruit and vegetable products, which causes the discoloration of the cut surface of fresh-cut fruit, catalyzes the oxidation of natural phenols to colorless quinone, and then polymerizes to produce color melanin-type chemical compounds [41]. As shown in Figure 6F, the activity of each group of PPO enzyme rose initially before decreasing, due to the mechanical damage of fresh-cut apples and the increase in PPO enzyme activity, which played a protective role. The control group reached the maximum on day 6, while the other treatment groups reached the maximum on day 8, indicating that the different components could delay the arrival of the peak PPO activity of the fresh-cut apple. Moreover, the PPO activity of each treatment group was lower than the control group throughout the storage period, revealing that the membrane treatment inhibited the PPO activity of the fresh-cut apple and decreased the degree of browning. The PPO enzyme activity is closely related to the degree of browning, and the SA-CW-G-Vc-Ca composite membrane liquid treatment group is essentially at the minimum level. On day 12, the PPO activity in the SA-CW-G-Vc-Ca composite membrane liquid treatment group was 25%, which was lower than the blank control treatment group and sodium alginate single membrane coating group, which determined it can significantly inhibit the browning degree of fresh-cut apples.

Since fresh-cut apples have no obvious tissue protection, they are susceptible to microbial attack and rot during storage [42]. Previous research has determined polysaccharide-based film treatment can effectively improve antimicrobial activity of fruits or vegetables, thus extending their shelf life [43]. The changes in the total number of colonies of fresh-cut apples treated with coating solutions of different components during storage at 4 °C were measured. In Figure 6G, the coating liquids of different components had a certain antibacterial effect on fresh-cut apples. On the 8th day, the total number of fresh-cut apple colonies in the blank control group (3.2 × 10^4^ CFU/m) was 8 times than that of the pure SA coating treatment (4 × 10^3^ CFU/mL), which was 24 times of the SA-CW-G-Vc-Ca composite membrane treatment group (1.3 × 10^3^ CFU/mL). On the 12th day, the total number of fresh-cut apple colonies treated with the SA-CW-G-Vc-Ca composite film solution was still the lowest, which indicated it can inhibit the reproduction of fresh-cut apple microorganisms. A study pointed out that the phenolic hydroxyl group of plant polyphenols could interact with carboxyl groups of polysaccharides. Thus, this kind of interaction can slow down the bioactive release from films, resulting in an improvement in the antimicrobial activity of the films. In this case, this hypothesis can explain why polysaccharide-based coatings can enhance the prevention of microbes, but the improvement of physical and mechanical properties provided by other additive ingredients are also necessary.

**Figure 7:**
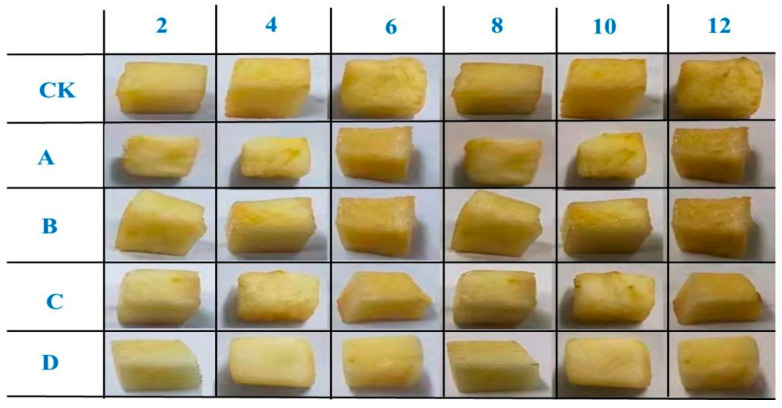
The shape and color changes of fresh-cut apples during 12 storage days (A: pure SA membrane; B:SA/CW composite membrane; C: SA/CW/G composite membrane; D: SA/CW/G/Vc-Ca composite membrane).

The shape and color changes of fresh-cut apples of different groups are displayed in Figure 7. As shown in Figure 6, it is apparent that the shape and color changes of fresh-cut apples coated with SA-CW-G-Vc-Ca composite membrane maintained the best quality during the storage days. This result coincided with the chemical measurements which determined the SA-CW-G-Vc-Ca composite membrane displayed the best protection ability for fresh-cut apples among the four films.

On the 8th day, the total number of fresh-cut apple colonies in the blank control group (3.2 × 10^4^ CFU/mL) was 8 times that of the pure sodium alginate coating treatment (4 × 10^3^ CFU/mL), which was the composite membrane treatment (1.3 × 10^3^).

On day 8, the colonies number of control fresh-cut apples group (3.2 × 10^4^ CFU/mL) has grown much more than the fresh cut apple coated by a pure sodium alginate (4 × 10^3^ CFU/mL) and a composite film (1.3 × 10^3^).

On the 12th day, the total number of fresh-cut apple colonies treated with the SA/CW/G/Vc-Ca composite film solution was still the lowest, which could inhibit the reproduction of fresh-cut apple microorganisms.

On day 12, the colony growth of fresh cut apples coated by a SA/CW/G/Vc-Ca composite membrane was still the lowest.

## 3. Materials and Methods

### 3.1. Experimental Materials

Sodium alginate (SA) of food grade was extracted and purified by Mingyue Seaweed Group Co. Ltd. (Shandong, China). Carnauba wax (CW) of food grade was purchased from Shanghai Yuanye Biotechnology Co. Ltd. (Shanghai, China). Calcium ascorbic acid (Vc-Ca) of food grade was purchased from Kangda Biology and Technology Co. Ltd. (Guangdong, China). Propanol of food grade was bought by Xilong Chemical Co. Ltd. (Shaanxi, China). Disodium hydrogen phosphate, sodium dihydrogen phosphate, and anhydrous calcium chloride (analytical pure) were obtained from Xilong Science Co. Ltd. (Shaanxi, China). Fresh apples were purchased in the market (Yang Ling Haoyouduo supermarket, Shaanxi, China) with neat shape, uniform size, maturity, and no obvious surface pests or appearance damage.

### 3.2. Preparation of the Edible Films

Sodium alginate powder (1 g) was dissolved into 100 mL deionized water. After it dissolved, 0.3 g carnauba wax was added, and the solution was heated and stirred until completely dissolved. Then 0.2 mL plasticizer glycerol was mixed into the solution, stirring evenly. When the temperature of the solution was approximately room temperature (25 °C), 0.4 g antioxidant calcium ascorbate was added to the solution, stirring until completely mixed. The film was obtained by pouring the film liquid in a plastic dish, and drying it in an oven at 50 °C [44,45].

The optimal formula of sodium alginate edible composite film, obtained through single factor and response surface design experiments in the early stage of this project, was: 1% sodium alginate, 0.3% carnauba wax, 0.2% glycerol, and 0.4% calcium ascorbate. In this article, the compositions of single or composite membranes were: A: pure SA membrane only containing 1% sodium alginate; B: SA-CW composite membrane composed of 1% sodium alginate and 0.3% carnauba wax; C: SA-CW-G composite membrane, composed of 1% sodium alginate, 0.3% carnauba wax, and 0.2% glycerol; D: SA-CW-G-Vc-Ca composite membrane composed of 1% sodium alginate, 0.3% carnauba wax, 0.2% glycerol, and 0.4% calcium ascorbate.

### 3.3. Performance of Film

#### 3.3.1. Determination of Water Vapor Transmittance

A 100 g anhydrous calcium chloride was placed in a small beaker, sealed with a membrane, and placed in an incubator at 25 °C and 75% relative humidity. After two days, the water vapor transmittance of different membranes was determined by the weight difference of anhydrous calcium chloride [45]. Calculation of film moisture permeability was:

WVP = m × d/(S × t × ΔP)(1)

In the formula, m is the mass increase in the weighing cup (g); d is film thickness (mm); S is the effective film area of water vapor transmission (m^2^); t is the determination time (d); the ΔP is the pressure difference between the upper and lower sides of the film (kPa); moisture permeability unit is g∙mm/ (cm^2^∙d∙KPa).

#### 3.3.2. Determination of Tensile Strength and Elongation at Break

Mechanical properties including tensile strength (TS) and elongation at break (E%) were performed by using a texture analyzer (TA.XT Plus, SMS Co., Godalming, UK). The initial distance is 20 mm, the maximum tensile force and the maximum tensile distance are set to 1000 g and 80 mm, and the test speed and post-test speed are 1 mm/s and 5 mm/s, respectively. The film was cut into 80 mm × 10 mm long strips for determination [46]. The calculations of the TS and E were completed through the following formulae:

TS = F/(L × W)(2)

The TS is tensile strength (MPa); F is the maximum tension of the specimen when it breaks (N); L is the thickness of the specimen (m); W is the width (m).

E = (L’ − L)/L(3)

The E is the elongation at break (%); the L’ is the length of the film when the sample is broken (m); L is the original length (m).

#### 3.3.3. Determination of Water Solubility

A certain weight of the film was added to 20 mL of distilled water, shaking evenly to make the film completely dissolve into the water, then sealed for 24 h. After filtration by Brinell funnel, the filter paper with insoluble substance was dried in an oven for 24 h. The dissolution rate of the edible film was calculated by the following formula:

WS = W_1_ − (W_3_ − W_2_)/W_1_ × 100 (4)

The WS is the solubility of the film (%); the W_1_ is the weight of the film (g); the W_2_ is the weight of filter paper (g); the W_3_ is the total mass of the dryer (g).

#### 3.3.4. Determination of Water Content

The composite film was stored in an oven of 100 °C until the composite film reached constant weight, and the water content in the composite film was measured. The average water content of each composite membrane is calculated repeatedly by using about 2 cm × 2 cm in three places of each membrane, and the water content of each sample is calculated by the following equation:

MC = (W_1_ − W_2_)/W_2_ (5)

The MC is moisture content (%); the W_1_ is film weight before drying (g); the W_2_ is film weight before drying (g).

### 3.4. Structure of Film Performance

#### 3.4.1. Scanning Electron Microscope (SEM) Analysis

A scanning electron microscope (S-3400N, Hitachi Limited Co., Tokyo, Japan) was used to analyze the ultrastructure of the film. In brief, the film was dried and cut into small pieces, mounted on the stub with adhesive carbon double-sided tape, coated with a platinum film under vacuum, and captured with an accelerating voltage of 15.0 kV. The samples were then scanned to obtain SEM micrographs.

#### 3.4.2. X-ray Diffraction Analysis

X-ray diffractometer (D8 ADVANCE A25, Bruker Co., Bremen, Germany) was used to operate under 40 kV and 40 mA under CuKα radiation 0.154 nm. The samples were scanned from 5° to 50° (2θ) at a speed of 2/min and a step size of 0.02°.

#### 3.4.3. Fourier Transform Infrared Spectroscopy (FT-IR) Analysis

The FT-IR spectra of edible membranes were determined by using a FT-IR spectrometer (MPA, Bruker Co., German). In attenuated total reflectance (ATR) mode, each spectrum was collected from 4000 to 400 cm^−1^. The air was used as the background scan.

### 3.5. Fresh-Cut Apple Coating Treatment

Apples were washed with ultrapure water and cut into 2 cm cubes after peeling and removing the core. The apple cubes were submerged in pure SA membrane solution (A), SA-CW composite membrane solution (B), SA-CW/G composite membrane solution (C), SA-CW-G/Vc-Ca composite membrane solution (D) for 3 min each. Distilled water (CK) was used as the blank control. After that, the cubes were placed into fresh-keeping bags and stored at 4 °C. Their color, hardness, weight loss rate, soluble solid, titratable acid, polyphen oloxidase activity, total colonies and other indicators were measured every 2 d.

### 3.6. Physiological and Biochemical Indexes of Fresh-Cut Apples

#### 3.6.1. Determination of Hardness

The hardness value of apple samples was measured using a texture analyzer. Test parameters: the pre-pressure speed is 1.0 mm/s, lower pressure speed is 0.5 mm/s, rising speed of 1.0 mm/s after pressure and the distance is 5 mm.

#### 3.6.2. Determination of Color

The color change was determined with a colorimeter at room temperature. The degree of browning was analyzed with the L* value and measured every 2 days [47]. The index was measured by the colorimeter, the L* value is the luminance index, which varies from 0~100, L* = 0 means black, while L* = 100 means white.

#### 3.6.3. Determination of Weight-Loss Rate

Direct weighing: weight-loss rate = (weight of pre-storage fruits−weight of fruit after storage time)/weight of pre-storage fruits [40].

#### 3.6.4. Determination of Titrable Acid

The content of titratable acid was determined by acid-base titration. Briefly, the treated apple cubes were added 30 mL distilled water for grinding. After filtering, 10 mL solution with 2–3 drops of Phenolphthale was titrated by 0.1 mol/L NaOH solution. When the solution became pink and does not fade after 30 s, the amount of NaOH solution was recorded. The content of titratable acid was calculated by the following formula:

TTA (%) = (A × 0.1 × C × K)/(W × D) × 100%(6)

In the formula, A is the NaOH quantity consumed (mL); C is the total diluted amount (mL); K is the malic acid conversion coefficient, 0.067; W is the sample weight (g); and D is the measured sampling quantity (mL).

#### 3.6.5. Determination of Soluble Solid-Form Content

A 100g of fresh-cut apple samples were ground in a mortar. After filtering, the soluble solid-form content of filtrate was measured with a refractometer.

#### 3.6.6. Determination of Polyphenol Oxidase Activity

Taking the increase in absorbance value of each gram of fruit and vegetable sample by 1 per minute as an activity unit, the unit is ΔOD_420_/min∙g. A certain amount of fruit was added buffer solution and ground evenly. After centrifugation at 12000× *g* at 4 °C for 30 min, the supernatant, the enzyme solution, was measured for its activity with an ultraviolet spectrophotometer (UV-1800, Mapada Co., Shanghai, China).

#### 3.6.7. Determination of Total Colonies

An appropriate amount of sample was added phosphate buffer to make a homogenous sample solution. After carrying out a 10-fold serial dilution, 1 mL diluted solution was pipetted onto a petri dish, then mixed. The number of corresponding colonies was recorded after culturing in an incubator at 37 °C for 72 h [24].

### 3.7. Statistical Analysis

All data in this study were measured at least three times and expressed as mean ± SD by analyzing for variance (ANOVA) followed with LSD multiple-range test. SPSS version 22.0 was used for all statistical analysis and the statistical significance of all experiments was defined as *p* < 0.05.

## 4. Conclusion

In this study, on the bases of conventional polysaccharide and lipids-based films, we added glycerin as a plasticizer to enhance the flexibility of the film and vitamin C as antioxidant, to enhance the antioxidant and anti-browning ability of the film. In addition, according to a properties comparison of different composite films and application on the preservation of fresh-cut apples, we obtained a new composite film with better physical and mechanical properties and performance on preservative effect on fresh-cut apples.

Although it is currently difficult to replace traditional plastic packaging with edible film, its advantages of high food safety and environmental friendliness establishes its development potential in the future. Compared to traditional packaging materials, the most important disadvantages of edible films are high solubility and low mechanical strength. However, by combining them with plasticizers, lipids, cross-linkers or other biological macromolecular substances, an edible film with ideal physical, mechanical and thermal properties could be obtained. In this study, sodium alginate was combined with carnauba wax, glycerin, and calcium ascorbate to obtain an edible film with better performance. Moreover, its formulation was optimized and its properties and structure were characterized. However, this study lacks a comprehensive determination on the properties of edible film and a deep investigation on film-forming solutions, such as the effects of different additives on viscosity, thermodynamic properties, elasticity, etc. For the application of edible film, this study determined the composite film had good preservative effect on fresh-cut apples by evaluating preservation indicators. However, there remains problems with practical applications. Because of the different physiological characteristics of various fruits and vegetables, specific fruit and vegetable varieties need corresponding films with specific preservation formulas. Although this study was not able to explore these aspects in detail, it can provide a theoretical basis for further study on edible film and a new way to engineer food packaging.

## Data Availability

Not applicable.

## Supplementary Materials

The following supporting information can be downloaded at: https://www.mdpi.com/article/10.3390/molecules28010367/s1, Table S1: Original data of Figure 2 and Figure 6.

## Abbreviations

SA	sodium alginate
CW	carnauba wax
G	glycerol
Vc-Ca	calcium ascorbic acid
